# Genetic, lifestyle, and health-related characteristics of adults without celiac disease who follow a gluten-free diet: a population-based study of 124,447 participants

**DOI:** 10.1093/ajcn/nqaa291

**Published:** 2020-11-12

**Authors:** Thomas J Littlejohns, Amanda Y Chong, Naomi E Allen, Matthew Arnold, Kathryn E Bradbury, Alexander J Mentzer, Elizabeth J Soilleux, Jennifer L Carter

**Affiliations:** Nuffield Department of Population Health, University of Oxford, Oxford, United Kingdom; The Wellcome Centre for Human Genetics, University of Oxford, Oxford, United Kingdom; Nuffield Department of Population Health, University of Oxford, Oxford, United Kingdom; British Heart Foundation Cardiovascular Epidemiology Unit, Department of Public Health and Primary Care, University of Cambridge, Cambridge, United Kingdom; School of Population Health, University of Auckland, Auckland, New Zealand; The Wellcome Centre for Human Genetics, University of Oxford, Oxford, United Kingdom; Department of Pathology, University of Cambridge, Cambridge, United Kingdom; Nuffield Department of Population Health, University of Oxford, Oxford, United Kingdom; NIHR Oxford Biomedical Research Centre, Oxford, United Kingdom

**Keywords:** gluten free, lifestyle, health, genome-wide association study, UK Biobank, cross-sectional study

## Abstract

**Background:**

The number of gluten-free diet followers without celiac disease (CD) is increasing. However, little is known about the characteristics of these individuals.

**Objectives:**

We address this issue by investigating a wide range of genetic and phenotypic characteristics in association with following a gluten-free diet.

**Methods:**

The cross-sectional association between lifestyle and health-related characteristics and following a gluten-free diet was investigated in 124,447 women and men aged 40–69 y from the population-based UK Biobank study. A genome-wide association study (GWAS) of following a gluten-free diet was performed.

**Results:**

A total of 1776 (1.4%) participants reported following a gluten-free diet. Gluten-free diet followers were more likely to be women, nonwhite, highly educated, living in more socioeconomically deprived areas, former smokers, have lost weight in the past year, have poorer self-reported health, and have made dietary changes as a result of illness. Conversely, these individuals were less likely to consume alcohol daily, be overweight or obese, have hypertension, or use cholesterol-lowering medication. Participants with hospital inpatient diagnosed blood and immune mechanism disorders (OR: 1.62; 95% CI: 1.18, 2.21) and non-CD digestive system diseases (OR: 1.58; 95% CI: 1.42, 1.77) were more likely to follow a gluten-free diet. The GWAS demonstrated that no genetic variants were associated with being a gluten-free diet follower.

**Conclusions:**

Gluten-free diet followers have a better cardiovascular risk profile than non-gluten-free diet followers but poorer self-reported health and a higher prevalence of blood and immune disorders and digestive conditions. Reasons for following a gluten-free diet warrant further investigation.

See corresponding editorial on page 491.

## Introduction

A gluten-free diet involves excluding foods containing wheat, rye, and barley and is the recommended treatment for celiac disease (CD), an autoimmune condition with a global prevalence of ∼1% (1). Although the prevalence of CD has remained stable in recent years, a US-based study found that the prevalence of gluten-free diet followers without a CD diagnosis more than tripled from 0.5% to 1.7% between 2009 and 2014 (2). This is reflected in market trends, which valued the global gluten-free products market at $4.2 billion in 2017, with a forecasted increase to $6.5 billion by 2023 (3).

Despite this increase in popularity, little is known about the characteristics of gluten-free diet followers without a CD diagnosis compared with the general population. Two studies, 1 based in the US (4) and 1 based in France (5), reported that gluten-free diet followers without a CD diagnosis were more likely to be women, have a higher education, have a higher household income, and have a lower BMI (in kg/m^2^). Although the findings are consistent with the perception that followers of a gluten-free diet tend to be better educated and generally healthier, the studies were small, consisting of only 155 (4) and 375 (5) gluten-free diet followers without a CD diagnosis, and they only investigated a limited range of characteristics.

Furthermore, genome-wide association studies (GWAS) have found that genetic factors are associated with intake of specific foods and beverages as well as dietary patterns (6, 7). However, to our knowledge, no GWAS has included following a gluten-free diet as an outcome. The identification of genetic factors could provide an insight into any potential biological mechanisms that influence the likelihood of following a gluten-free diet. For instance, human leukocyte antigen (HLA) haplotypes DQ2 and DQ8 are a necessary cause of CD, triggering an abnormal autoimmune response through interaction with gluten peptides (8). Other non-HLA variants have been implicated in CD susceptibility, and it is plausible that these, along with HLA DQ2 and DQ8, could be associated with the avoidance of gluten in those without CD through similar mechanisms (9).

To address these issues, we investigated the association between a wide range of sociodemographic, lifestyle, physical, and health-related characteristics and following a gluten-free diet in a United Kingdom–based cohort of ∼125,000 participants without CD. We also performed a GWAS to investigate whether any genetic factors are associated with following a gluten-free diet.

## Methods

### Population

UK Biobank (UKB) is a population-based cohort study that recruited half a million women and men aged 40–69 y (10). All participants attended 1 of 22 baseline assessment centers located in England, Scotland, and Wales between 2006 and 2010. At baseline, participants provided sociodemographic, lifestyle, and health-related information through a touchscreen questionnaire and verbal interview, underwent a range of physical measures, and provided blood samples. All participants provided electronic signed consent to take part in UKB and for UKB to perform ongoing linkage to health-related records.

UKB received approval from the National Information Governance Board for Health and Social Care and the National Health Service North West Centre for Research Ethics Committee (Ref: 11/NW/0382).

### Assessment of gluten-free status

Gluten-free status was ascertained through the Oxford WebQ, an online questionnaire in which participants reported their dietary intake from the previous 24 h using a list of 206 preselected foods and beverages (11). The Oxford WebQ was incorporated into the baseline assessment between 2009 and 2010 and was completed by ∼70,000 participants. The Oxford WebQ was subsequently completed online by ∼176,000 participants on ≤4 further occasions between 2011 and 2012 (12). As part of the questionnaire, participants were asked, “Do you routinely follow a special diet?” with 1 of the options being “Gluten free or wheat free diet.” Participants were identified as following a gluten-free diet if they responded as such on ≥2 questionnaires.

### Assessment of sociodemographic, lifestyle, physical, and health-related characteristics

Townsend deprivation score was used as a measure of socioeconomic status and was assigned to participants based on their residential postcode at recruitment (13). Region was derived from assessment center location. Information on ethnicity, education, alcohol intake, smoking status, physical activity, weight change in the past year, major dietary change in the past 5 y, use of medication (antihypertensive, cholesterol-lowering, insulin, analgesic, and constipation or heartburn medication), overall health status, and the presence of a long-standing illness was self-reported through the touchscreen questionnaire. Amounts of physical activity (low, moderate, and high) were derived in accordance with the International Physical Activity Questionnaire guidelines (14). BMI was derived from weight using scales and standing height measured during the physical examination. Systolic and diastolic blood pressure was measured twice, with ≥1 min between measurements, using an Omron 705 IT electronic blood pressure monitor with the participant in a seated position. For the current analysis, a mean of the 2 readings was derived, with participants categorized as either “normal” (systolic ≤140 mm Hg and diastolic ≤90 mm Hg) or “hypertensive” (systolic >140 mm Hg or diastolic >90 mm Hg, or were taking antihypertensive medication).

### Diagnoses from medical records

Diagnoses of prevalent disease at baseline were recorded using the International Classification of Diseases, 10th revision (ICD-10), coding system and were ascertained using hospital inpatient and cancer registry records available for all participants. The hospital inpatient records were obtained from Hospital Episode Statistics for England (1996 onward), Scottish Morbidity Record for Scotland (1981 onward), and Patient Episode Database for Wales (1998 onward). The cancer registry records were obtained from NHS Digital for England and Wales (1971 onward) and the NHS Central Register for Scotland (1957 onward). Primary diagnoses were extracted from the hospital inpatient records for ICD-10 chapters A, B, D–N, R–T, and Z, whereas codes C00–C97 (except C44—nonmelanoma skin cancer) were extracted from the cancer registry records. Participants were classified as having a prevalent diagnosis for each ICD chapter if they had a diagnosis in the relevant chapter prior to the baseline assessment.

### GWAA

The generation of genetic data in UKB has been described in detail elsewhere (15). A GWAS with imputed genetic data was carried out using linear mixed models as implemented in SAIGE (version 0.29.1) adjusting for age and genetic sex (16).

### Statistical analysis

Multivariable logistic regression models were used to assess the association between sociodemographic, lifestyle, physical, health-related characteristics, and prevalent diagnoses by ICD-10 chapter and the probability of following a gluten-free diet. All models were adjusted for age, sex, Townsend deprivation score (quintiles), education [no qualifications, lower secondary (CSE/O-Level/GCSE or equivalent), upper secondary (AS/A-Level or equivalent), higher education, or other professional qualification], ethnicity (white, nonwhite), smoking (never, former, current), alcohol (never drinker, former drinker, special occasions only, 1–3 times a month, 1–2 times a week, 3–4 times a week, daily, or almost daily), physical activity (low, moderate, high), and BMI (<18.5, ≥18.5 to <25, 25 to <30, ≥30), with the exception of the sociodemographic models, which were adjusted for age and sex only. Bonferroni correction was applied to the prevalent diagnoses analyses to account for multiple comparisons (17 tests).

Analyses were performed using STATA SE version 15 (StataCorp), and figures were produced using R version 3.6.1 (R Foundation for Statistical Computing).

## Results

**FIGURE 1:**
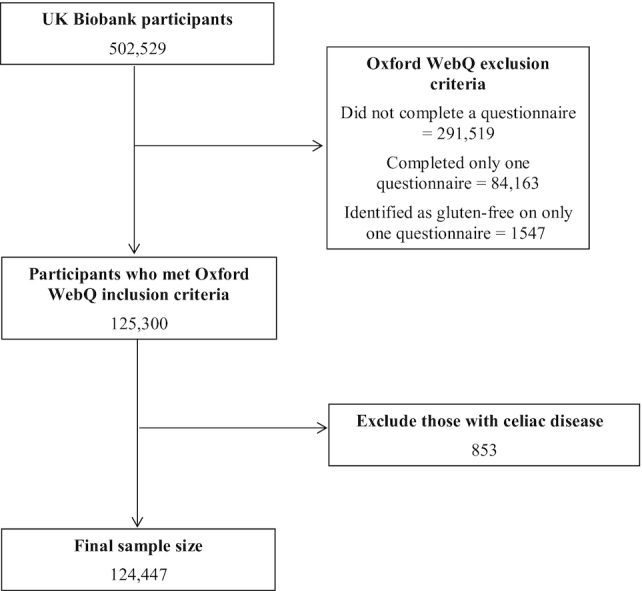
Flowchart for final analytic sample size.

Among 502,529 participants, 211,010 completed ≥1 Oxford WebQ. Of these, 84,163 participants who only completed 1 questionnaire were excluded, as well as 1547 participants who identified themselves as following a gluten-free diet only once out of the five 24-h assessments. A further 853 participants who were identified as having CD through self-report at baseline and/or hospital inpatient records (available up to March 2017 for England, October 2016 for Scotland, and February 2016 for Wales; diagnosed using ICD-10 code K90.0, ICD-9 code 579.0) were excluded, resulting in a final analytical sample of 124,447 participants (**Figure 1**).

Among the 124,447 participants included in the current analyses, 1776 (1.4%) reported following a gluten-free diet.

The associations of sociodemographic characteristics with gluten-free diet followers without a CD diagnosis are shown in **Table 1**. In fully adjusted models, age was not associated with following a gluten-free diet (OR for 60–70 y compared with 40–49 y: 0.96; 95% CI: 0.85, 1.09), whereas women had almost 4-fold higher OR of following a gluten-free diet compared with men (age-adjusted OR: 3.92; 95% CI: 3.47, 4.44). Furthermore, participants of nonwhite ethnic origin, who lived in more socioeconomically deprived areas, and who had a higher level of education were more likely to follow a gluten-free diet. There was also substantial regional variation, with following a gluten-free diet being most common in London and South England and least common in the Midlands, North England, and Wales.

**TABLE 1:** Association between sociodemographic characteristics and following a gluten-free diet

	Gluten-free diet		
Sociodemographic characteristics	No, *n* (%)	Yes, *n* (%)	OR^1^ (95% CI)^2^
Age group, y			
40–49	28,794 (23.5)	464 (26.1)	1 (Ref)
50–59	44,026 (35.9)	685 (38.6)	1.03 (0.91, 1.16)
60–70	49,851 (40.6)	627 (35.3)	0.96 (0.85, 1.09)
Women	67,587 (55.1)	1471 (82.8)	3.92 (3.47, 4.44)
Nonwhite ethnic origin	3714 (3.0)	100 (5.7)	1.53 (1.24, 1.89)
Region			
London	24,825 (20.2)	493 (27.8)	1 (Ref)
South England	24,022 (19.6)	437 (24.6)	1.10 (0.96, 1.27)
Wales	3861 (3.2)	45 (2.5)	0.71 (0.52, 0.97)
Midlands	17,389 (14.2)	181 (10.2)	0.62 (0.52, 0.74)
North England	46,062 (37.6)	525 (29.6)	0.69 (0.61, 0.79)
Scotland	6512 (5.3)	95 (5.4)	0.84 (0.67, 1.06)
Townsend deprivation score			
Least deprived (<–2)	67,954 (55.5)	863 (48.6)	1 (Ref)
Average (–2 to 1.99)	39,232 (32.0)	595 (33.5)	1.12 (1.00, 1.25)
Most deprived (≥2)	15,340 (12.5)	317 (17.9)	1.47 (1.28, 1.70)
Education			
No qualifications	7993 (6.5)	66 (3.7)	1 (Ref)
Lower secondary	16,855 (13.8)	206 (11.6)	1.26 (0.96, 1.68)
Upper secondary	7724 (6.3)	104 (5.9)	1.40 (1.03–1.92)
Higher education or other professional qualification or equivalent	89,714 (73.4)	1393 (78.8)	1.76 (1.37–2.26)

1 ORs were calculated using logistic regression.

2 Models adjusted for age, sex, ethnicity, region, Townsend deprivation score, and education.

Fully adjusted associations of lifestyle and physical characteristics with gluten-free diet followers without a CD diagnosis are shown in **Table 2**. Participants who consumed more alcohol and who had a higher BMI were less likely to follow a gluten-free diet. Conversely, participants who were never drinkers, former drinkers, former smokers, and who had lost weight in the previous year were more likely to follow a gluten-free diet. Making dietary changes due to illness was associated with more than a 6-fold higher OR of following a gluten-free diet (OR: 6.35; 95% CI: 5.60, 7.20). Physical activity, gaining weight in the past year, and current smoking were not significantly associated with following a gluten-free diet.

**TABLE 2:** Association between lifestyle and physical characteristics and following a gluten-free diet^1^

	Gluten-free diet		
Lifestyle and physical characteristics	No, *n* (%)	Yes, *n* (%)	OR^2^ (95% CI)^3^
Alcohol consumption			
Never	3434 (2.8)	99 (5.6)	1.75 (1.40, 2.20)
Former	3451 (2.8)	132 (7.4)	2.57 (2.11, 3.15)
Special occasions only	11,187 (9.1)	261 (14.7)	1.43 (1.22, 1.67)
1–3 times a month	13,150 (10.7)	234 (13.2)	1.17 (0.99, 1.37)
1–2 times a week	30,102 (24.6)	429 (24.2)	1 (Ref)
3–4 times a week	31,746 (25.9)	359 (20.2)	0.81 (0.70, 0.93)
Daily or almost daily	29,530 (24.1)	261 (14.7)	0.65 (0.56, 0.77)
Smoking status			
Never	69,999 (57.2)	1031 (58.2)	1 (Ref)
Former	43,852 (35.8)	641 (36.2)	1.22 (1.10, 1.35)
Current	8551 (7.0)	101 (5.7)	0.93 (0.76, 1.15)
IPAQ group for physical activity			
Low	18,749 (18.2)	263 (17.7)	1 (Ref)
Moderate	44,340 (43.1)	632 (42.5)	0.97 (0.84, 1.12)
High	39,883 (38.7)	593 (39.9)	1.03 (0.88, 1.19)
BMI, kg/m^2^			
Underweight (<18.5)	671 (0.6)	29 (1.6)	1.74 (1.19, 2.55)
Normal weight (18.5–24.9)	47,488 (38.8)	865 (48.9)	1 (Ref)
Overweight (25–29.9)	50,168 (41.0)	564 (31.9)	0.75 (0.68, 0.84)
Obese (≥30)	24,067 (19.7)	312 (17.6)	0.74 (0.65, 0.84)
Weight change in past year			
No	72,093 (59.6)	980 (55.9)	1 (Ref)
Gained weight	30,394 (25.1)	456 (26.0)	1.03 (0.92, 1.16)
Lost weight	18,551 (15.3)	318 (18.1)	1.17 (1.03, 1.34)
Dietary change in past 5 y			
No	78,138 (63.8)	613 (34.6)	1 (Ref)
Yes, due to illness	9875 (8.1)	491 (27.7)	6.35 (5.60, 7.20)
Yes, other reason	34,537 (28.2)	669 (37.7)	2.34 (2.10, 2.62)

1 IPAQ, International Physical Activity Questionnaire.

2 ORs were calculated using logistic regression.

3 Models adjusted for age, sex, ethnicity, Townsend deprivation score, education, smoking, alcohol, physical activity, and BMI.

Fully adjusted associations of health-related characteristics with gluten-free diet followers without a CD diagnosis are shown in **Table 3**. Participants with hypertension or who used cholesterol-lowering medication were less likely to follow a gluten-free diet. In contrast, use of medications for constipation or heartburn was associated with a higher likelihood of following a gluten-free diet, whereas there were no significant associations with use of insulin or analgesic medications. Compared with participants who reported their overall health as good, those who reported their overall health as fair (OR: 1.31; 95% CI: 1.16, 1.49) or poor (OR: 2.10; 95% CI: 1.68, 2.63) were more likely to follow a gluten-free diet, whereas those who rated their overall health as excellent were less likely to do so (OR: 0.66; 95% CI: 0.57, 0.75). Participants with a self-reported long-standing illness were also more likely to follow a gluten-free diet (OR: 1.82; 95% CI: 1.64, 2.01).

**TABLE 3:** Association between health-related characteristics and following a gluten-free diet

	Gluten-free diet		
Health-related characteristics	No, *n* (%)	Yes, *n* (%)	OR^1^ (95% CI)^2^
High blood pressure or using antihypertensives	58,317 (47.5)	592 (33.3)	0.70 (0.63, 0.78)
Using cholesterol-lowering medication	18,268 (14.5)	120 (6.8)	0.56 (0.46, 0.67)
Using insulin	922 (0.8)	9 (0.5)	0.76 (0.39, 1.47)
Using analgesic medications	43,343 (35.6)	642 (36.4)	1.02 (0.92, 1.12)
Using medications for constipation/heartburn	10,045 (8.3)	222 (12.6)	1.52 (1.32, 1.76)
Overall health rating			
Excellent	26,674 (21.8)	276 (15.6)	0.66 (0.57, 0.75)
Good	73,211 (59.8)	1059 (59.9)	1 (Ref)
Fair	19,384 (15.8)	338 (19.1)	1.31 (1.16, 1.49)
Poor	3165 (2.6)	95 (5.4)	2.10 (1.68, 2.63)
Long-standing illness	34,594 (28.7)	703 (40.8)	1.82 (1.64, 2.01)

1 ORs were calculated using logistic regression.

2 Models adjusted for age, sex, ethnicity, Townsend deprivation score, education, smoking, alcohol, physical activity, and BMI.

**FIGURE 2:**
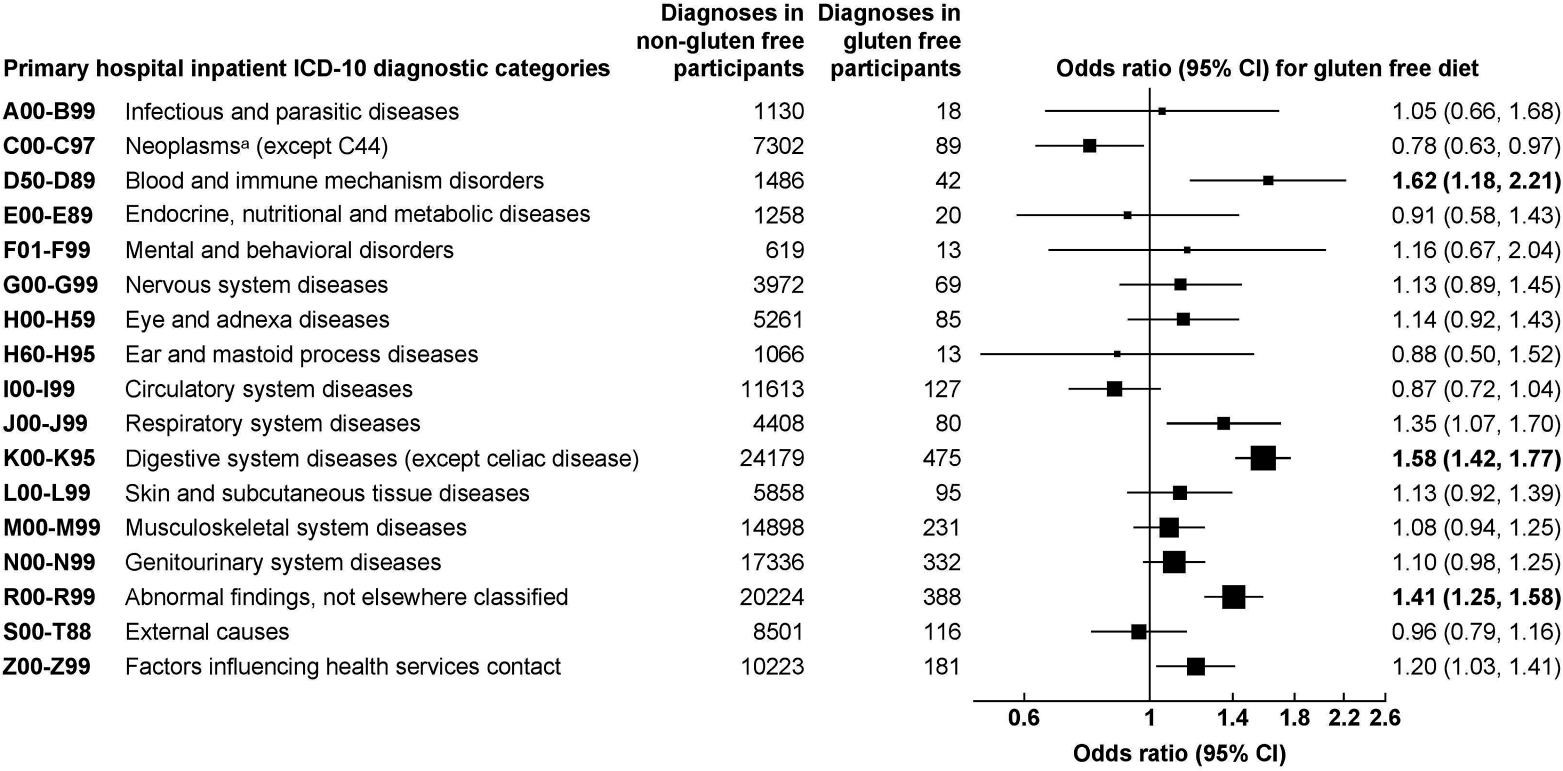
Association of hospital inpatient diagnoses by ICD-10 chapter with following a gluten-free diet. Models are adjusted for age, sex, ethnicity, Townsend deprivation score, education, smoking, alcohol, physical activity, and BMI. Information on cancers was obtained from cancer registry data and not hospital inpatient records. Bold values indicate associations that are significant at the Bonferroni corrected level *P* < 0.003 (allowing for 17 tests). ICD-10, International Classification of Diseases, 10th revision.

After allowing for multiple testing, participants with prevalent hospital inpatient diagnoses of blood and immune mechanism disorders (ICD-10 chapter D50–D89; OR: 1.62; 95% CI: 1.18, 2.21) and non-CD digestive system diseases (ICD-10 chapter K00–K95; OR: 1.58; 95% CI: 1.42, 1.77) were more likely to follow a gluten-free diet (**Figure 2**). Participants with symptoms, signs, and abnormal clinical and laboratory findings, not elsewhere classified, were also more likely to follow a gluten-free diet (ICD-10 chapter R00–R99; OR: 1.41; 95% CI: 1.25, 1.58). Regarding the previously mentioned ICD-10 chapters, the following conditions had a higher prevalence in participants following a gluten-free diet: nutritional, aplastic, and other anaemias; gastrointestinal conditions; and symptoms and signs involving the digestive system (**Supplemental Table 1**). No other significant associations between prevalent hospital inpatient diagnoses by ICD-10 chapter and following a gluten-free diet were observed.

**FIGURE 3:**
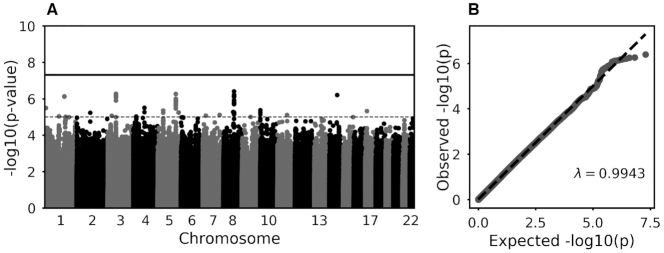
Manhattan (A) and QQ plot (B) of GWAS of gluten-free diet with age and sex as covariates. The solid line indicates genome-wide significance; the dashed line indicates suggestive significance. GWAS, genome-wide association study.

In the GWAS, no loci reached genome-wide significance (**Figure 3**). Summary statistics for single nucleotide polymorphisms that met the sub-GWAS threshold for significance are provided in **Supplemental Table 2**.

## Discussion

This is the first large population-based study to investigate the genetic, lifestyle, and health-related characteristics of individuals without CD who follow a gluten-free diet. With regard to sociodemographic factors, gluten-free diet followers were more likely to be women, nonwhite, and have higher levels of education. For lifestyle and physical characteristics, following a gluten-free diet was associated with being a former or never drinker, a former smoker, having a lower BMI, having lost weight in the past year, and having made major dietary changes in the past 5 y as a result of illness. With regard to health-related characteristics, cardiovascular risk factors, including high blood pressure and use of lipid-lowering medication, were associated with lower odds of following a gluten-free diet; however, reporting fair or poor health, a long-standing illness, or having a hospital inpatient diagnosis for blood or digestive disorders was associated with higher odds of following a gluten-free diet. A GWAS found no significant associations with following a gluten-free diet, providing no evidence for a specific genetic predisposition to this dietary preference.

To date, there has been a lack of research investigating the characteristics of gluten-free diet followers. A US-based study reported similar findings to those observed in UKB, with gluten-free diet followers (*n* = 155) more likely to be women, have a higher level of education, and experiencing significant weight loss during the past year (4). A French-based study reported both similar and contrasting findings with those observed in UKB, with gluten-free diet followers (*n* = 375) more likely to be women, older, never-smokers, and less educated (5). These conflicting findings indicate that some characteristics associated with following a gluten-free diet in people without CD may be population specific. Although we observed that nonwhite participants were more likely to follow a gluten-free diet, we were unable to investigate this in more specific ethnic subgroups due to the small percentage of UKB participants who are nonwhite (17). Studies in diverse populations with larger sample sizes are necessary to investigate these sociodemographic and lifestyle associations further.

In the current study, some of the characteristics of participants following a gluten-free diet suggest a “healthier” phenotype—for example, lower BMI, less likely to be hypertensive, less likely to use cholesterol-lowering medications, and lower alcohol consumption. Despite this, gluten-free diet followers had poorer self-reported health; were more likely to have a long-standing illness; and had a higher prevalence of hospital-diagnosed digestive, blood, and immune mechanism disorders. It is possible that individuals with pre-existing health conditions, particularly digestive disorders, could choose to follow a gluten-free diet in order to address health concerns. This is supported by the finding that gluten-free diet followers were >6 times more likely to report making dietary changes in the past 5 y as a result of illness. Furthermore, gluten-free diet followers were more likely to report making other lifestyle changes, such as being former drinkers and former smokers. In the current study, it is not possible to infer the reasons for following a gluten-free diet or to determine whether following a gluten-free diet occurred before or after the onset of health issues or lifestyle changes. Both qualitative and quantitative research will need to elucidate the reasons for following a gluten-free diet—if the choice was recommended by a clinician or self-guided—and, if the choice was self-guided, to investigate what sources are used to inform the decision to follow a gluten-free diet.

There has been limited research investigating the association between gluten intake and subsequent health outcomes. In >100,000 US-based adults, higher gluten intake was associated with a reduced risk of incident coronary heart disease over >20 y of follow-up when adjusting for intake of refined grains (18). In a separate US-based study of ∼200,000 adults, a higher gluten intake was associated with a decreased risk of incident type-2 diabetes over 28 y, whereas a lower gluten intake was related to a reduced consumption of fibers and other beneficial nutrients (19). The nutrient quality of gluten-free diets has consistently been shown to be poorer compared with that of traditional diets (20, 21), and consequently this could increase the risk of certain health outcomes. In the current study, we found that gluten-free diet followers had poorer health. Future longitudinal studies investigating incident disease in gluten-free diet followers need to consider prevalent disease because this may contribute to the decision to follow a gluten-free diet and also affect the risk of subsequent disease.

This is the first study to investigate whether genetic factors are associated with following a gluten-free diet, with no variants reaching genome-wide level of significance. This included a lack of association in regions known to be implicated in CD, which suggests that the mechanisms driving gluten avoidance in CD are not shared with those who avoid gluten without CD. However, these findings should be considered preliminary due to the low prevalence of gluten-free diet cases (1.4%) in UKB, which limits the detection of small effect sizes (22), as well as the lack of replication in an independent population. Several variants reached subgenome-wide significance and warrant further investigation once genetic data sets with phenotyping for gluten-free diet have been established.

A limitation of the current study is its cross-sectional design, which prevents inferences about temporality regarding the uptake of a gluten-free diet and disease or other risk factors, such as smoking and drinking, that may affect disease risk. We attempted to restrict the study to participants without CD by excluding those who self-reported CD at baseline or had a prevalent or incident hospital diagnosis of CD. Nevertheless, it is possible that some of the gluten-free diet followers had not yet been diagnosed with CD or had a diagnosis in another setting (i.e., primary care). The self-reported health behaviors and medication use may also have been subject to reporting bias; however, there is no clear indication that those following a gluten-free diet were more likely to be influenced by reporting bias given that some, but not all, medication use was associated with lower odds of following a gluten-free diet and that self-reported poor health was associated with higher odds of following a gluten-free diet. We also lacked information about the length of time people had followed a gluten-free diet; nevertheless, to be classified as following a gluten-free diet, participants had to have self-reported this on ≥2 occasions. Furthermore, the response rate of participants recruited into UKB was low (5.5%), with a further decline in response for those who responded to the online WebQ survey of dietary intake (17). The prevalence of those reporting gluten-free diets in UKB is therefore not representative of the United Kingdom, although importantly, the associations between participant characteristics and following a gluten-free diet should be less affected by this selection bias (17). Despite lacking in representativeness, UKB is the largest study to report on gluten-free diet followers without a known diagnosis of CD, which allowed for the first time a reliable investigation into a variety of genetic and phenotypic characteristics as well as linkage with hospital inpatient records to determine prevalent diagnoses.

This comprehensive study reports a wide range of sociodemographic, lifestyle, and health factors associated with following a gluten-free diet in those without a diagnosis of CD—a growing demographic. Our results suggest that gluten-free diet followers have some attributes that may confer a lower risk of cardiovascular disease but also a higher likelihood of certain prevalent diseases and long-standing illnesses. Future research exploring the underlying reasons for choosing to follow a gluten-free diet without a diagnosis of CD is warranted.

## Supplementary Material

nqaa291_Supplemental_File

## Notes

This work was supported by core grants to the Clinical Trial Service Unit from the Medical Research Council (JLC; Clinical Trial Service Unit grant A310) and the British Heart Foundation (JLC; grant CH/1996001/9454) and by the NIHR Oxford Biomedical Research Centre (JLC). UK Biobank was established by the Wellcome Trust, Medical Research Council, Department of Health, and the Scottish Government. UK Biobank has also received funding from the Welsh Assembly Government, British Heart Foundation, and Diabetes UK. The research was designed, conducted, analyzed, and interpreted by the authors independently of funding agencies.

Supplemental Tables 1 and 2 are available from the “Supplementary data” link in the online posting of the article and from the same link in the online table of contents at https://academic.oup.com/ajcn/.

Data described in the manuscript, code book, and analytical code will be made available upon request pending approval by UK Biobank. UK Biobank is an open access resource. Bona fide researchers can apply to use the UK Biobank data set by registering and applying at http://ukbiobank.ac.uk/register-apply. All results presented in this manuscript, including the code used to generate them, will be returned to UK Biobank within 6 mo of publication, at which point they are made available for researchers to request (subject to UK Biobank approval).

Abbreviations used: CD, celiac disease; GWAS, genome-wide association study; HLA, human leukocyte antigen; ICD-10, International Classification of Diseases, 10th revision; UKB, UK Biobank.
