# Supplementary material for: Genetic, lifestyle, and health-related characteristics of adults without celiac disease who follow a gluten-free diet: a population-based study of 124,447 participants
Source: Am J Clin Nutr. 2020 Nov 12;113(3):622–9. doi: 10.1093/ajcn/nqaa291 (PMC7948866; doi:10.1093/ajcn/nqaa291)
Supplement: nqaa291_Supplemental_File [file nqaa291_supplemental_file.docx]

Genetic, lifestyle and health-related characteristics of adults without celiac disease who follow a gluten-free diet: a population-based study of 124,453 participants. Thomas J. Littlejohns, et al.

Online Supplementary Material

**Supplementary Table 1** – Hospital inpatient diagnoses in ICD-10 chapters D, K and R by gluten-free diet status

| **ICD-10 primary diagnoses** | **Gluten-free** | | **p-value^1^** |
| --- | --- | --- | --- |
|  | **No**  **N (%)** | **Yes**  **N (%)** |  |
| **Chapter D – blood and immune mechanism disorders** |  |  |  |
| **50-53** – nutritional anaemias | 494 (0.4) | 18 (1.0) | <0.001 |
| **55-59** – haemolytic anaemias | 17 (0.01) | 1 (0.06) | 0.14 |
| **60-64** – aplastic and other anaemias | 638 (0.5) | 17 (1.0) | 0.01 |
| **65-69** - coagulation defects, purpura and other haemorrhagic conditions | 94 (0.08) | 1 (0.06) | 0.76 |
| **70-77** - other diseases of blood and blood-forming organs | 273 (0.2) | 6 (0.3) | 0.31 |
| **80-89** - certain disorders involving the immune mechanism | 86 (0.07) | 2 (0.11) | 0.50 |
| **Chapter K – digestive system diseases** |  |  |  |
| **00-14** - diseases of oral cavity, salivary glands and jaws | 2,746 (2.2) | 40 (2.3) | 0.97 |
| **20-31** - diseases of oesophagus, stomach and duodenum | 7,486 (6.1) | 159 (9.0) | <0.001 |
| **35-38** - diseases of appendix | 728 (0.6) | 10 (0.6) | 0.87 |
| **40-46** - hernia | 5,163 (4.2) | 60 (3.4) | 0.08 |
| **50-52** - noninfective enteritis and colitis | 2,355 (1.9) | 94 (5.3) | <0.001 |
| **55-64** - other diseases of intestines | 7,416 (6.1) | 168 (9.5) | <0.001 |
| **65-67** - diseases of peritoneum | 132 (0.1) | 7 (0.4) | <0.001 |
| **70-77** - diseases of liver | 222 (0.2) | 6 (0.3) | 0.13 |
| **80-87** - disorders of gallbladder, biliary tract and pancreas | 2,644 (2.2) | 33 (1.9) | 0.39 |
| **90-93** - other diseases of the digestive system (except coeliac disease) | 728 (0.6) | 24 (1.4) | <0.001 |
| **Chapter R – abnormal findings not elsewhere classified** | | | |
| **00-09** - symptoms and signs involving the circulatory and respiratory systems | 6,222 (5.1) | 97 (5.5) | 0.46 |
| **10-19** - symptoms and signs involving the digestive system and abdomen | 6,527 (5.3) | 195 (11.0) | <0.001 |
| **20-23** - symptoms and signs involving the skin and subcutaneous tissue | 600 (0.5) | 9 (0.5) | 0.92 |
| **25-29** - symptoms and signs involving the nervous and musculoskeletal systems | 194 (0.2) | 4 (0.2) | 0.48 |
| **30-39** - symptoms and signs involving the urinary system | 3,709 (3.0) | 46 (2.6) | 0.29 |
| **40-46** - symptoms and signs involving cognition, perception, emotional state and behaviour | 401 (0.3) | 9 (0.5) | 0.19 |
| **47-49** - symptoms and signs involving speech and voice | 147 (0.1) | 3 (0.2) | 0.55 |
| **50-69** - general symptoms and signs | 4,420 (3.6) | 84 (4.7) | 0.01 |
| **70-79** - abnormal findings on examination of blood, without diagnosis | 571 (0.5) | 5 (0.3) | 0.26 |
| **80-82** - abnormal findings on examination of urine, without diagnosis | 41 (0.03) | 1 (0.06) | 0.60 |
| **83-89** - abnormal findings on examination of other body fluids, substances and tissues, without diagnosis | 362 (0.3) | 5 (0.3) | 0.92 |
| **90-94** - abnormal findings on diagnostic imaging and in function studies, without diagnosis | 485 (0.4) | 12 (0.7) | 0.06 |

^1^ Calculated using chi-square

**Supplementary Table 2** – Summary statistics for sub-GWAS significant SNPs

| **Region** | **Lead SNP** | **Effect allele** | **Effect allele frequency** | **Odds Ratio**  **(95% CI)** | ***p*-value** |
| --- | --- | --- | --- | --- | --- |
| 1:9565028-10649054 | rs74925829 | T | 0.018547065 | 1.864 (1.434, 2.422) | 3.24E-06 |
| 1:111040652-111047669 | rs868170376 | A | 0.225468556 | 0.828 (0.762, 0.900) | 9.34E-06 |
| 1:152918837-153156898 | rs55983724 | G | 0.039313099 | 1.569 (1.312, 1.875) | 7.48E-07 |
| 1:163195713-163343813 | rs60467814 | TAA | 0.444293693 | 1.163 (1.088, 1.244) | 9.84E-06 |
| 2:105818980-105895307 | rs114993523 | A | 0.040055949 | 1.549 (1.282, 1.873) | 5.95E-06 |
| 3:30539127-30552292 | 3:30551032_TATA_T | T | 0.17382115 | 0.803 (0.729, 0.884) | 8.69E-06 |
| 3:62955857-63143030 | rs12487147 | C | 0.440271786 | 1.187 (1.110, 1.269) | 5.40E-07 |
| 4:22813713-23278752 | rs550904028 | TTA | 0.018254539 | 0.591 (0.468, 0.746) | 9.50E-06 |
| 4:87058183-87868011 | rs17450910 | A | 0.012374147 | 2.142 (1.555, 2.950) | 3.17E-06 |
| 5:41427388-42826419 | rs114754636 | G | 0.026396778 | 1.675 (1.344, 2.087) | 4.49E-06 |
| 5:140146894-140629690 | rs6887594 | C | 0.228986408 | 0.815 (0.753, 0.883) | 5.55E-07 |
| 5:162404568-162626996 | rs2964354 | T | 0.457150303 | 1.165 (1.091, 1.245) | 5.74E-06 |
| 7:22812578-23102329 | rs10263435 | A | 0.227411961 | 1.199 (1.107, 1.299) | 8.57E-06 |
| 7:130112557-130421159 | rs71581008 | A | 0.462309774 | 1.176 (1.095, 1.262) | 7.80E-06 |
| 8:76543188-76966378 | rs118095218 | G | 0.014713165 | 2.013 (1.489, 2.721) | 5.38E-06 |
| 8:83730221-83811692 | rs1997054 | G | 0.789017341 | 0.809 (0.745, 0.878) | 3.97E-07 |
| 10:1010596-1302040 | rs117838935 | G | 0.012429582 | 2.158 (1.555, 2.995) | 4.28E-06 |
| 11:74161461-74257582 | rs686622 | G | 0.707286606 | 1.18 (1.098, 1.270) | 7.88E-06 |
| 14:80891386-80954070 | rs76232800 | T | 0.06843708 | 1.438 (1.247, 1.659) | 6.32E-07 |
| 17:12758843-12848327 | rs201609903 | C | 0.29249037 | 0.843 (0.784, 0.907) | 4.79E-06 |

Abbreviations: CI, Confidence Interval, GWAS, Genome Wide Association, SNP, Single Nucleotide Polymorphism
